# Perceived Self Efficacy in Implementing COVID-19 Preventive Measures Among Residents of Harari Regional State, Eastern Ethiopia: A Community-Based Cross-Sectional Study

**DOI:** 10.3389/fepid.2022.849015

**Published:** 2022-04-25

**Authors:** Addis Eyeberu, Adera Debella, Dechasa Adare Mengistu, Mesay Arkew, Saba Hailu, Amanuel Oljira, Jemal Yusuf Kebira, Tamirat Getachew, Shamble Nigussie, Deribe Bekele, Rebuma Belete, Degu Abate, Habtamu Bekele, Tigist Tefera, Addisu Alemu, Yadeta Dessie

**Affiliations:** ^1^School of Nursing and Midwifery, College of Health and Medical Sciences, Haramaya University, Harar, Ethiopia; ^2^Department of Environmental Health, College of Health and Medical Science, Haramaya University, Harar, Ethiopia; ^3^Department of Medical Laboratory Sciences, College of Health and Medical Sciences, Haramaya University, Harar, Ethiopia; ^4^School of Public Health, College of Health and Medical Sciences, Haramaya University, Harar, Ethiopia; ^5^School of Pharmacy, College of Health and Medical Sciences, Haramaya University, Harar, Ethiopia; ^6^Biochemistry Department, School of Medicine, College of Health and Medical Sciences, Haramaya University, Harar, Ethiopia

**Keywords:** community, COVID-19, preventive measure, novel coronavirus, Ethiopia

## Abstract

**Background:**

The COVID-19 outbreak has now become a major international public health concern and a major challenge for the entire world. Poor adherence to COVID-19 prevention measures continues to be a challenge in managing COVID-19 pandemics, including in Ethiopia. As a result, the current study sought to identify the determinants of community adherence to COVID-19 preventive measures among the adult population of Harari Regional State in Eastern Ethiopia.

**Methods:**

A community-based cross-sectional study was conducted in Harari Regional State, Eastern Ethiopia from January 5 to 30, 2021. All adults above the age of 18 who lived in Harari Regional State's selected kebeles were eligible to participate in the study. A systematic random sampling method was used to select the study participants. The study included a total of 1,320 participants. Pre-tested, structured, and self-administered questionnaires were used to collect data, which was then analyzed using STATA version 16 software. The consent was obtained from each individual and concerned body. Binary logistic regression was used to find the correlation between community adherence to COVID-19 preventive measures and sociodemographic, knowledge, self-efficacy, and risk perception. A *P*-value of 0.05 was used as the statistical significance cut-off point.

**Results:**

One thousand two hundred fifty-five (1,255) people took the survey, yielding a 95.1 % response rate. Adherence to COVID-19 preventive measures resulted in a mean cumulative score of 29.8. Six hundred eight (48.5%) of the participants had good adherence to COVID-19 preventive measures, whereas 647 (51.5%) did not. The researchers also discovered a statistically significant link between participants' residence, educational status, risk perception, income, and adherence to COVID-19 preventive measures.

**Conclusion:**

Appropriate implementation of COVID-19 preventive measures among community members is required to manage or control pandemics and reduce health-related consequences associated with COVID-19 pandemics. The current study, on the other hand, discovered that nearly half of the study participants had poor adherence. As a result, in order to manage this pandemic, the relevant organizations, including the government and non-governmental organizations, must take appropriate and timely measures.

## Introduction

The novel-coronavirus disease is currently a global health threat and an international public health emergency. In 2012, another coronavirus-caused outbreak known as Middle Eastern Respiratory Syndrome (MERS) resulted in over 1,000 infections and 400 deaths through 2015, and now the new Coronavirus disease 2019 (COVID-19) has plagued the world (1, 2). COVID-19 is a newly emerging disease caused by a highly infectious novel coronavirus that primarily affects the respiratory system (3). COVID-19 was first detected and isolated from a pneumonia patient in late December 2019 from Wuhan, China. Then WHO declared COVID-19 as a pandemic on 11 March 2020 (4). The most common symptoms include fever (78%), cough (57%), and fatigue (31%), as well as severe viral pneumonia, which can lead to acute respiratory distress syndrome, which can be fatal. COVID-19 can be transmitted to humans by touching an infected object or surface and then touching their mouth, nose, or possibly their eyes (4, 5). Both the CDC and WHO recommend that coronavirus prevention measures be known and implemented. These urge countries around the world to prioritize strict non-pharmaceutical interventions to combat the pandemic, such as the (mandatory) use of face masks, physical separation, regular hand washing and use of sanitizers, and regularly disinfecting surfaces (6).

Ethiopia is the second most populous nation in Africa, with a 9% of the African population (7). Since COVID-19 was declared a global and national public health threat, the Ethiopian government has implemented additional preventive measures such as mandatory quarantine periods for all travelers, restrictions on public gatherings, school closures, and mandatory facemasks in public places, as well as fewer passengers using public transportation. Risk communication on measures such as physical separation, wearing face masks, and hygiene, including through the media and cell-phone ring tone reminders, are critical interventions, but the infection rate continues to rise (8).

Despite government-imposed restrictions, there are inconsistencies in the implementation of COVID-19 preventive measures. Low educational status, female gender, age, urban residence, and knowledge of COVID 19 were discovered to have a significant effect on adherence to COVID preventive measures (6, 9, 10).

Poor adherence to COVID-19 mitigation measures remains a problem (11). Understanding the level of adherence to non-pharmaceutical interventions is essential to control COVID-19 disease (6). However, there is no adequate and current evidence regarding community adherence to COVID-19 preventive measures. Thus, the current study aimed to determine and provide current evidence on the community's adherence toward COVID-19 preventive measures and root causes of poor adherence among residents of Harari Region, Eastern Ethiopia.

## Materials and Methods

### Study Period, Design, and Setting

A community-based cross-sectional study was conducted in Harari regional state, Eastern Ethiopia, from January 5 to 30, 2021. The region is located 510 kilometers east of Addis Ababa, Ethiopia's capital city. The region is divided into nine districts, three of which are rural and the other six are urban. According to the 2021 projection based on the 2007 Census, CSA, the region's total population is 270,000, with 136,000 males and 134,000 females (1). The Harari regional state had a total of 59,487 households. During the survey period, the information regarding COVID-19 has been disseminated by the concerned organizations, including the regional health bureau. However, there was a gap in the implementation of COVID-19 prevention measures with an unknown magnitude or level.

### Study Population

All adult residents of Harari Regional State who are above the age of 18 and live in selected kebeles (Smallest administrative unit in Ethiopia). The study included residents living in selected households as heads or any other household members over the age of 18 who were available during data collection. People who left the house for any reason and became seriously ill during data collection were barred from participating in the study.

### Sample Size Determination and Sampling Procedures

The sample size was determined using the single population proportion formula by considering the expected margin of error (d) of 0.04, with a confidence interval level of 95% and a design effect of 2. The acceptance rate of preventive measures taken from a similar study was 48.96% (11). The number of households to be included in this study was calculated based on the following formula.


$$\begin{array}{l} N\;=\;\frac{2{\left(z\right)}^{2}x\;p\;\left(1-p\right)}{d^{2}} \end{array}$$

$N\;=\;\frac{2{\left(1.96\right)}^{2}x\;0.4896\;\left(1\;-\;0.4896\right)}{0.04^{2}}$ = 1200. Then, the final sample size required for the current study was 1,320, including 10% non-response rate.

The study participants were sampled using a multi-stage sampling technique. The simple random sampling (lottery) method was used to select 13 kebeles from 9 districts. One thousand and three hundred and twenty households were allocated proportionally from the selected kebeles. A systematic random sampling method was used to select each study participant (HH). Then, a total of 1,320 eligible individuals were chosen from the included households, with 1,255 completing the survey.

### Data Collection Methods and Techniques

A face-to-face interview was used to collect data using a pre-tested, semi-structured questionnaire (with three sections that include socio-demographic characteristics, preventive behaviors, and self-efficacy to practice COVID-19). Investigators used an online survey portal to create the questionnaires, and Google form was used to collect the data.

Thirty data collectors (BSc holders) and 8 supervisors (MSc. holders) were trained on the ways of data collection, principles, and ethics before data collection. The questionnaire was adapted from the WHO and UNICEF documents of COVID-19 preparedness and response (12), and similar literature (3, 11, 13).

### Measurement

Adherence to COVID-19 prevention methods was measured using 10 questions. The respondents rated how often they were following the preventive methods recommended by WHO on five scales: none (1), rarely (2), sometimes (3), frequently (4), and always (5). Finally, the cumulative practice score was computed (range 10–50). Finally, a higher score presents good adherence to preventive against COVID-19 (above mean score), whereas the inverse represents poor adherence (14).

Perceived self-efficacy to practice prevention measures was measured using four items which responded on five scales: certainly not (1), probably not (2), not perhaps yes (3), probably yes (4), and most certainly (5). The items were stated in a way that the higher value indicates more perceived self-efficacy to practice the measures. The sum and mean scores were computed. Finally, those who had a higher score presented high self-efficacy to practice COVID-19 prevention measures based on the mean score (above mean score), whereas the inverse represent low self-efficacy (3).

### Data Quality Control

Prior to data collection, the questionnaire was pretested in Haramaya Town, Eastern Ethiopia, outside the study area, to ensure the clarity, sequence, and applicability of questions, as well as to estimate the time required to collect data. For three days, all data collectors and supervisors were thoroughly trained on the principles, ethics, procedures, and questionnaire. All completed questionnaires were reviewed for consistency and completeness.

### Statistical Processing and Analysis

The data were checked for consistency and completeness. The data were analyzed using STATA version 16 software. Descriptive statistical tests were used to provide a clear distribution of the data. Numerical variables were measured as mean and standard deviations, while categorical variables were expressed as frequencies and percentages. Finally, binary logistic regression was applied to examine the effects of independent variables on adherence toward COVID-19 preventive measures and it was expressed as an odds ratio (OR) and 95% confidence interval (CI). A *P*-value of 0.05 was considered as a cut-off point for statistical significance.

## Results

### Socio-Demographic Characteristics

The survey was completed by 1,255 people out of a total of 1,320 sample sizes, yielding a 95.1% response rate. The average age of respondents was 34.4 (SD 10.9) years old, with nearly one-fourth being between the ages of 18 and 25.0 (Table 1).

**Table 1:** Socio-demographic characteristics of study participants in Harari Regional State, Eastern Ethiopia 2021 (*N* = 1,255).

**Variables** **(*N* = 1,255)**	**Categories**	**Frequency**	**Percentage (%)**
Age group (years)	18-25	304	24.3
	26-35	478	38.1
	35-45	302	24.1
	>45	171	13.6
Sex	Male	634	50.5
	Female	621	49.5
Marital status	Single	220	17.5
	Married	923	73.6
	Divorced/widowed	112	8.9
Educational status	No formal education	358	28.5
	Primary education	399	31.8
	Secondary education	254	20.2
	Collage and above	244	19.4
Residence	Urban	776	61.8
	Rural	479	38.2
Income in ETB	<5,000	1,018	81.2
	5,000-10,000	215	17.2
	>10,000	22	1.8

*ETB, Ethiopian Birr*.

### Adherence to COVID-19 Preventive Measures

About one-fourth (24.4%) of the study participants were never maintaining physical distance, whereas only 3.9% keep their distance always. About one-fourth (24.4%) of study participants were never maintained physical distance, whereas only 3.9% always maintained physical distance.

Eleven percent (11%) of the respondents never wear face masks outside the home and around 12% of participants always wash their hands with water and soap. The study found the mean cumulative score of adherences to COVID-19 preventative measure that was 29.8 (SD ± 7.8. Six hundred eight, 608 (48.5%) had good adherence to COVID 19 preventive measures while 647 (51.5%) had a poor adherence (Table 2).

**Table 2:** Adherence to COVID 19 preventive measures among adult population in Harari Regional State, Eastern Ethiopia, 2021.

**Statements (*N* = 1,255)**	**Never**		**Rarely**		**Sometimes**		**Frequently**		**Always**	
	***N***	**%**	***N***	**%)**	***N***	**%**	***N***	**%**	***N***	**%**
How often are you maintaining physical distance?	306	24.4	435	34.5	382	30.5	83	6.6	49	3.9
How often are you avoiding larger gatherings?	328	26.5	464	36.9	351	27.9	80	6.8	32	2.6
How often are you avoiding touching your face, eyes, mouth, and nose?	265	21.1	381	30.5	393	31.3	168	13.4	48	3.8
How often are you washing your hands with water and soap or sanitizers?	101	8.1	269	21.4	462	36.8	273	21.8	150	11.9
How often are you avoiding contact with people who had fever and cough?	154	12.3	312	24.9	319	25.4	255	20.3	215	17.1
How often are you wearing a facemask when you are at work or outside the home?	150	11.9	280	22.3	456	36.3	230	18.3	139	11.1
How often do you use public transportation during the months of the pandemic?	467	37.2	318	25.4	314	25.02	126	10.1	30	2.4
How often you avoid unprotected contacting (touching) of frequently contacted surfaces?	311	24.8	358	28.5	412	32.8	130	10.4	44	3.5
How often are you staying home to prevent COVID-19 infection?	451	35.9	371	29.7	276	21.9	100	8.0	57	4.5
How often are you wearing a glove at work?	719	57.3	212	16.9	187	14.9	90	7.2	47	3.8

### Risk Perception Regarding COVID-19

One-third (33.6%) of the study participants agreed that getting sick with the coronavirus can be dangerous. Regarding preventive measures, about 81.0% of respondents believe that they will contract coronavirus if they do not take any preventive measures. The mean cumulative score of risk perception was 29.8 (SD 6.9). Five hundred eighty-eight, 588(46.9%) of the participants had a high-risk perception toward COVID-19, whereas 667(53.2%) had a low-risk perception (Table 3).

**Table 3:** Risk perception toward COVID-19 pandemic among adult population in Harari Regional State, Eastern Ethiopia, 2021 (*N* = 1,255).

**Statements** **(*N* = 1,255)**	**Category**	**Frequency**	**Percentage (%)**
New coronavirus risk is very dangerous.	Strongly agree	370	29.5
	Agree	442	33.6
	Neutral	246	19.6
	Disagree	163	12.9
	Strongly disagree	54	4.30
I think that I am likely to become sick with the new coronavirus.	Strongly agree	414	32.9
	Agree	604	48.2
	Neutral	151	12.1
	Disagree	9	0.7
	Strongly disagree	77	6.2
I think that COVID-19 causes more deaths than other respiratory diseases.	Strongly agree	573	45.7
	Agree	495	39.5
	Neutral	96	7.7
	Disagree	73	5.8
	Strongly disagree	18	1.4
I think that work exposes me more to COVID-19.	Strongly agree	604	48.1
	Agree	426	33.9
	Neutral	146	11.6
	Disagree	48	3.8
	Strongly disagree	31	2.5
I think getting sick with the coronavirus can be serious.	Strongly agree	593	45.8
	Agree	517	39.9
	Neutral	94	7.3
	Disagree	74	5.7
	Strongly disagree	18	1.4
I think my health will be severely damaged if you contract coronavirus.	Strongly agree	411	32.8
	Agree	536	42.7
	Neutral	121	9.6
	Disagree	133	10.6
	Strongly disagree	54	4.3
I think it is not possible to recover from coronavirus disease.	Strongly agree	148	11.8
	Agree	299	23.8
	Neutral	139	11.1
	Disagree	483	38.5
	Strongly disagree	186	14.8
I think If I caught with coronavirus, I cannot manage your daily activities.	Strongly agree	323	25.7
	Agree	534	42.6
	Neutral	173	13.8
	Disagree	200	15.9
	Strongly disagree	25	2
I think people may stigmatize me if I get sick due to coronavirus.	Strongly agree	275	21.9
	Agree	480	38.3
	Neutral	221	17.6
	Disagree	225	17.9
	Strongly disagree	54	4.3
I think that I will contract coronavirus if I do not take any preventive measures.	Strongly agree	385	30.7
	Agree	634	50.5
	Neutral	135	10.8
	Disagree	89	7.1
	Strongly disagree	12	0.9
I think that I will contract coronavirus if I take preventive measures.	Strongly agree	193	15.4
	Agree	414	32.9
	Neutral	161	12.8
	Disagree	394	31.4
	Strongly disagree	93	7.4
I think that I will contract coronavirus if I come into contact with a coronavirus patient.	Strongly agree	437	34.8
	Agree	655	52.2
	Neutral	108	8.6
	Disagree	45	3.6
	Strongly disagree	10	0.8
I think that I will contract coronavirus even if I do not come into contact with a coronavirus patient.	Strongly agree	243	19.4
	Agree	395	31.5
	Neutral	157	12.5
	Disagree	405	32.3
	Strongly disagree	55	4.4
I think that the coronavirus will not affect very many people in the area I am currently living.	Strongly agree	258	20.6
	Agree	471	37.5
	Neutral	237	18.9
	Disagree	236	18.8
	Strongly disagree	53	4.2

### Perceived Self-Efficacy to Implement COVID-19 Prevention Measures

Among 1,255 respondents, only 15.6% were certain that they could wash their hands with water and soap or with sanitizers frequently. Furthermore, about 17.2% of the study participants reported that they certainly did not manage to use facemasks outside the home. According to the current study, the average cumulative score of adherences to COVID-19 preventative measures was 29.8 (SD = 7.8).

The mean cumulative score of self-efficacy items was 11.9 (SD ± 3.9). Five hundred eighty (46.2%) of the participants had high self-efficacy to practice COVID-19 preventive methods, while the remaining 675 (53.78%) had a low self-efficacy to practice COVID-19 preventive methods (Table 4).

**Table 4:** Self-efficacy to practice COVID-19 prevention methods among adults in Harari region, East Ethiopia, 2021, (*N* = 1,255).

**Questions** **(*N* = 1,255)**	**Category**	**Frequency**	**Percentage (%)**
Do you think that you manage to hand wash with water and soap or sanitizer frequently?	Certain not	142	11.3
	Probably not	252	20.1
	Perhaps not perhaps	128	10.2
	Probably yes	537	42.8
	Certainly yes	196	15.6
Do you think that you manage to stay at home?	Certain not	244	19.4
	Probably not	358	28.5
	Perhaps not perhaps	200	15.9
	Probably yes	347	27.7
	Certainly yes	106	8.5
Do you think that you manage to maintain distancing anywhere?	Certain not	192	15.3
	Probably not	344	27.4
	Perhaps not perhaps	195	15.5
	Probably yes	406	32.4
	Certainly yes	118	9.40
Do you think that you manage to use a face-mask always outside the home or at work?	Certain not	215	17.1
	Probably not	332	26.5
	Perhaps not perhaps	186	14.8
	Probably yes	407	32.4
	Certainly yes	115	9.2

### Factors Associated With the Adherence of Adults Toward COVID-19 Safety Measures

The multivariate analysis shows that participants residing in rural areas were 0.71 times less likely to have poor adherence to COVID-19 preventive measures than their counterparts (AOR: 0.71, 95%CI: 0.52-0.98). The odds of poor adherence to safety measures of COVID-19 were 0.39 times less likely among adults who had primary education than respondents who did not attend formal education (AOR: 0.61, 95%CI: 0.43-0.87). Similarly, participants with a monthly income of 5,000-10,000 ETB were 0.59 times less likely to adhere poorly to the preventive measures (AOR: 0.41, 95%CI: 0.28-0.59) compared with those who had an income of <5,000 ETB. The odds of poor COVID-19 preventive behavior among respondents who had low perceived self-efficacy was almost four times higher than those who had high self-efficacy to practice COVID-19 prevention methods (AOR: 3.8, 95%CI: 3.01-4.97) (Table 5).

**Table 5:** Factors associated with poor adherence to COVID-19 preventive measures among adults in Harari Region, East Ethiopia, 2021 (*N* = 1,255).

**Variables (*N* = 1,255)**	**Adherence toward preventive measures**		**Crude OR**	**Adjusted OR**	***P*-value**
	**Poor**	**Good**			
**Age**					
18-25	152 (50)	152 (50)	1	1	
26-35	232 (48.5)	246 (51.5)	0.69 (0.79-1.41)	0.89-1.89	0.174
35-45	150 (49.6)	152 (50.4)	1.01 (0.74-1.39)	0.64-1.50	0.940
>45	74 (43.3)	97 (56.7)	1.3 (0.89-1.91)	0.68-1.85	0.631
**Sex of respondents**					
Male	326 (51.4)	308 (48.6)	1	1	
Female	339 (54.6)	282 (45.4)	1.2 (1.01-1.58)	0.812-1.39	0.616
**Resident**					
Urban	380 (49.4)	390 (50.6)	1	1	
Rural	267 (55.7)	212 (44.3)	1.3 (1.04-1.65)	0.71 (0.52-0.98)	0.046
**Marital status**					
Single	101 (45.9)	119(54.1)	1	1	
Married	485 (52.5)	438 (47.5)	1.3 (0.97-1.75)	0.96 (0.61-1.50)	0.860
Divorced/widowed	61 (54.5)	51 (45.5)	1.4 (0.89-2.23)	1.09 (0.59-2.018)	0.768
**Educational status**					
No formal education	227 (63.4)	131 (36.6)	1	1	
Primary school education	203 (50.8)	196 (49.2)	0.59 (0.44-0.79)	0.61 (0.43-0.87)	0.007
Secondary school education	119 (46.8)	135 (51.2)	0.5 (0.36-0.70)	0.7 (0.45-1.09)	0.117
Collage and above	98 (40.1)	146 (59.9)	0.38 (0.20-0.54)	1.06 (0.63-1.789)	0.805
**Occupation**					
Housewife	153 (60.2)	101 (39.8)	1	1	
Government employee	307 (58.2)	220 (41.8)	0.9 (0.67-1.24)	0.5 (0.29-0.84)	0.465
Private employee	62 (44.9)	76 (55.1)	0.5 (0.35-0.818)	0.86 (0.58-1.27)	0.010
Farmer	65 (30.2)	150 (69.8)	0.28 (0.19-0.42)	0.24 (0.13-0.43)	0.000
Student	60 (49.6)	61 (50.4)	0.64 (0.42-1.02)	0.61 (0.32-1.13)	0.118
**Income**					
<5,000	577 (56.7)	441 (43.3)	1	1	
5,000-10,000	63 (29.3)	152 (70.7)	0.31 (0.23-0.44)	0.41 (0.28-0.59)	0.000
>10,000	7	15	0.35 (0.14-0.88)	0.38 (0.14-1.01)	0.053
**Perceived self-efficacy**					
High	306 (52)	282 (48)	1	1	
Low	341(51.1)	326 (48.9)	3.8 (3.07-4.92)	3.8 (3.01-4.97)	0.000

## Discussion

Preventive measures play an essential role in reducing infection rates and controlling the spread of various diseases. There has been no definitive treatment found since the emergence of the COVID-19 pandemic. As a result, adhering to the recommended preventive measures is the best option for limiting the spread of the COVID-19 pandemic (11). The current study sought to investigate the community's adherence to COVID-19 prevention measures in Harari Regional State, Ethiopia. In the current study, 1,255 adults provided complete responses. According to the current study, overall poor adherence to COVID-19 preventive measures accounted for 51.5%, which was higher than the finding of another study conducted in Ethiopia, which reported a community adherence level of 44.1% to the recommended COVID-19 safety measures (15). But higher than the finding of another study conducted in Ethiopia, which found only 12.3% of the study participants adhered to the recommended COVID-19 preventive measures (16).

The current study identified various factors related to COVID-19 adherence among the study participants. For example, an educational status: primary school [AOR = 0.61 (0.43-0.87)] was associated with poor adherence to COVID-19 preventive measures that were in line with the findings reported by another study conducted in Ethiopia (15). Other factors associated with poor adherence to COVID-19 preventive measures were occupational status, monthly income, and perceived self-efficacy. The current study found that being a governmental employee [AOR = 0.5 (0.29-0.84] and formal occupations [AOR = 0.24 (0.13-0.43)] were significantly associated with poor adherence to the COVID-19 preventive measures. Another study conducted in Congo also reported that being both a private and public employee was associated with poor adherence to COVID-19 preventive measures (6). Even though the two studies' findings sound discrepant, they are almost identical in their findings except their interpretations.

The current study has also found that a monthly income of more than 5,000 ETB [AOR = 0.41 (0.28-0.59)] was significantly associated with poor adherence to the COVID-19 preventive measures. This finding was in line with the finding of another study conducted in Saudi Arabia and Mexico (16, 17).

Furthermore, the current study found a statistically significant association between perceived self-efficacy [AOR = 0.25 (0.20-0.32)] and poor adherence to COVID-19 preventive measures among the study participants. Another similar study conducted in Ethiopia reported that having no perceived efficacy was significantly associated with poor adherence to COVID-19 preventive measures (15). The discrepancy may be due to the socio-cultural differences between the two areas of communities.

Overall, the current study found more than half of the study participants had poor adherence to COVID-19 preventive measures. Therefore, to reduce these gaps and to increase the implementation of COVID-19 preventive measures, and manage the COVID-19 pandemic, the local and federal governments should design the appropriate intervention strategies based on the level of community understanding.

## Conclusion

Appropriate implementation of COVID-19 preventive measures among community members is required to manage or control COVID-19 pandemics and reduce health-related consequences associated with COVID-19 pandemics. The current study, on the other hand, discovered that less than half of the study participants had poor adherence. As a result, in order to manage this pandemic, the relevant organizations, including the government and non-governmental organizations, must take appropriate and timely measures.

## Limitations of the Study

A community-based cross-sectional study design was used, and data was collected at predetermined time intervals. As a result, cause and effect relationships could not be investigated.

## Data Availability Statement

The raw data supporting the conclusions of this article will be made available by the authors, without undue reservation.

## Ethics Statement

The studies involving human participants were reviewed and approved by Ethical clearance was taken from Institutional Health Research Ethics Review Committee (IHRERC) of Haramaya University. In addition, permission letters were obtained from all the concerned organization/bodies and from participants. The patients/participants provided their written informed consent to participate in this study.

## Author Contributions

AE conceived the idea and had major roles in the data review, drafting, and editing the manuscript. All authors contributed to data analysis, drafting and revising the manuscript, and have read and approved the final version of the manuscript to be published and agreed on all aspects of this work.

## Conflict of Interest

The authors declare that the research was conducted in the absence of any commercial or financial relationships that could be construed as a potential conflict of interest.

## Publisher's Note

All claims expressed in this article are solely those of the authors and do not necessarily represent those of their affiliated organizations, or those of the publisher, the editors and the reviewers. Any product that may be evaluated in this article, or claim that may be made by its manufacturer, is not guaranteed or endorsed by the publisher.

## Abbreviations

CDC: Center for Disease Control and Prevention
COVID-19, Coronavirus disease: 2019
CSA: Center of Statistical Agency
ETB: Ethiopian Birr
HH: Household
MERS: Middle Eastern Respiratory Syndrome
SD: Standard Deviation
UNICEF: United Nations Children's Fund
WHO: World Health Organization.

## References

[1] Lango MN. How did we get here? Short history of COVID-19 and other coronavirus-related epidemics. Head Neck. (2020) 42:1535–8. 10.1002/hed.26275 32445249 PMC7283747

[2] Adela N Nkengazong L Ambe LA Ebogo JT Medou Mba F Goni HO. Knowledge, attitudes, practices of/towards COVID 19 preventive measures and symptoms: a cross-sectional study during the exponential rise of the outbreak in Cameroon. PLoS Negl Trop Dis. (2020) 14:e0008700. 10.1371/journal.pntd.0008700 32886678 PMC7497983

[3] Asefa A Qanche Q Hailemariam S Dhuguma T Nigussie T. Risk perception towards COVID-19 and its associated factors among waiters in selected towns of southwest Ethiopia. Risk Manag Healthc Policy. (2020) 13:2601. 10.2147/RMHP.S276257 33223860 PMC7671469

[4] Grant MC Geoghegan L Arbyn M Mohammed Z McGuinness L Clarke EL. The prevalence of symptoms in 24,410 adults infected by the novel coronavirus (SARS-CoV-2; COVID-19): a systematic review and meta-analysis of 148 studies from 9 countries. PLoS ONE. (2020) 15:e0234765. 10.1371/journal.pone.0234765 32574165 PMC7310678

[5] Omemo P Wasonga J. Determinants of adherence to the recommended COVID-19 Prevention and Control Guidelines by Small Scale Retail Shop Operators in Rural Parts of Siaya County, Kenya. J Epidemiol Public Health Rev. (2020) 5. 10.16966/2471-8211.198

[6] Ditekemena JD Nkamba DM Muhindo HM Fodjo Siewe JN Luhata C Van den Bergh R. Factors associated with adherence to COVID-19 prevention measures in the Democratic Republic of the Congo (DRC): results of an online survey. BMJ Open. (2021) 11:e043356. 10.1136/bmjopen-2020-043356 33462101 PMC7813390

[7] Breevoort A Carosso GA Mostajo-Radji MA. High-altitude populations need special considerations for COVID-19. Nat Commun. (2020) 11:1–3. 10.1038/s41467-020-17131-6 32612128 PMC7329858

[8] Deressa W Worku A Abebe W Getachew S Amogne W. Social distancing and preventive practices of government employees in response to COVID-19 in Ethiopia. PLoS ONE. (2020) 16:e0257112. 10.1371/journal.pone.0257112 34492089 PMC8423289

[9] Bante A Mersha A Tesfaye A Tsegaye B Shibiru S Ayele G. Adherence with COVID-19 preventive measures and associated factors among residents of Dirashe District, Southern Ethiopia. Patient Prefer Adher. (2021) 15:237. 10.2147/PPA.S293647 33568900 PMC7868191

[10] Weiss BD Paasche-Orlow MK. Disparities in adherence to COVID-19 public health recommendations. Health Lit Res Pract. (2020) 4:e171–3. 10.3928/24748307-20200723-01 32926173 PMC7410493

[11] Azene ZN Merid MW Muluneh AG Geberu DM Kassa GM Yenit MK. Adherence towards COVID-19 mitigation measures and its associated factors among Gondar City residents: a community-based cross-sectional study in Northwest Ethiopia. PLoS ONE. (2020) 15:e0244265. 10.1371/journal.pone.0244265 33378332 PMC7773181

[12] UNICEF. RCCE Action Plan Guidance COVID-19 Preparedness and Response. Gonder city: UNICEF (2019).

[13] Asnakew Z Asrese K Andualem M. Community risk perception and compliance with preventive measures for COVID-19 pandemic in Ethiopia. Risk Manag Healthc Policy. (2020) 13:2887–97. 10.2147/RMHP.S279907 33335434 PMC7737628

[14] Wolff K Larsen S Øgaard T. How to define and measure risk perceptions. Annals Tour Res. (2019) 79:102759. 10.1016/j.annals.2019.102759

[15] Qanche Q Asefa A Nigussie T Hailemariam S Duguma T. Knowledge of COVID-19 and preventive behaviors among waiters working in food and drinking establishments in Southwest Ethiopia. PLoS ONE. (2021) 16:e0245753. 10.1371/journal.pone.0245753 33493226 PMC7833477

[16] Al-Hanawi MK Angawi K Alshareef N Qattan AMN Helmy HZ Abudawood Y. Knowledge, attitude and practice toward COVID-19 among the public in the Kingdom of Saudi Arabia: a cross-sectional study. Front Public Health. (2020) 8:217. 10.3389/fpubh.2020.00217 32574300 PMC7266869

[17] Irigoyen-Camacho ME Velazquez-Alva MC Zepeda-Zepeda MA Cabrer-Rosales MF Lazarevich I Castaño-Seiquer A. Effect of income level and perception of susceptibility and severity of COVID-19 on stay-at-home preventive behavior in a group of older adults in Mexico City. Int J Environ Res Public Health. (2020) 17:7418. 10.3390/ijerph17207418 33053788 PMC7601258

